# Implementation and Resource Optimization of an Oral Anticancer Medication Clinical Pharmacy Trainee Program: Operational and Financial Impact at a Tertiary Cancer Centre

**DOI:** 10.3390/curroncol33090523

**Published:** 2026-08-31

**Authors:** Christine Peragine, Flay Charbonneau, Susan Singh, Carlo DeAngelis

**Affiliations:** 1 Department of Pharmacy, Sunnybrook Odette Cancer Centre, Toronto, ON M4N 3M5, Canada; 2 Leslie Dan Faculty of Pharmacy, University of Toronto, Toronto, ON M5S 3M2, Canada

**Keywords:** oral anticancer medications, clinical pharmacy services, clinical key performance indicators, pharmacy education, experiential training, health economics, workforce optimization

## Abstract

Background: The emergence of oral anticancer medications (OAMs) has increased demand for specialized clinical pharmacy services (CPSs). Concurrently, Canadian cross-sectional data highlights undergraduate education gaps, with fewer than 14% of community pharmacists reporting adequate training on OAM therapies. We designed, implemented, and evaluated a pharmacist-led Oral Anticancer Medication Clinical Co-op Program (OAMCCP) to bridge this experiential education gap while cost-effectively expanding institutional capacity. Methods: Launched in Fall 2022, the OAMCCP integrated one PharmD student per 16-week term into a multidisciplinary oncology specialty pharmacy. Trainees executed core ambulatory oncology clinical pharmacy key performance indicators (AOcpKPIs), including Best Possible Medication Histories (BPMHs), drug–drug interaction (DDI) screenings, and proactive adherence and toxicity follow-up. Student competency was verified by a licensed oncology pharmacist to maintain patient safety. Impact was quantified via self-reported task logging over 15 weeks, standardized 10-point patient satisfaction surveys, payroll expenditure comparisons, and student testimonials. Results: Trainees completed 673 clinical tasks (~45 tasks/week), contributing 16.2 h of direct clinical support weekly—effectively adding +0.43 full-time equivalent (FTE) to service capacity. Mean patient satisfaction was 9.5/10 (*n* = 29), with students successfully managing inquiries in 96.5% of encounters. Financially, a 1.0 FTE student (44,700 CAD/year cost) captured clinical capacity valued at 69,300 CAD/year (0.43 FTE pharmacist equivalence). Conclusions: The OAMCCP resolves experiential training gaps while presenting a safe, scalable, and financially viable human resource framework that expands oncology pharmacy services.

## Simple Summary

The rapid growth in the number of oral anticancer medications is placing mounting operational and financial strain on outpatient hospital budgets, as the intensive clinical monitoring required to ensure patient safety demands the continuous expansion of licensed clinical pharmacist staffing—a model that is increasingly unsustainable within public healthcare systems. This study evaluates an innovative solution: embedding pharmacy co-op students into structured, algorithm-driven ambulatory care workflows at a comprehensive Canadian cancer centre. Over the evaluation period, the trainee resource safely absorbed over 600 complex clinical tasks and captured 43% of a full-time licensed pharmacist’s workload capacity. By leveraging supervised pharmacy trainees, the program expanded institutional workload capacity equivalent to a clinical pharmacist at approximately 65% of the total fiscal investment. This operational framework provides cancer centres with a blueprint to increase pharmacy capacity, optimize labor expenses, and actively contain institutional costs without compromising patient care.

## 1. Introduction

The landscape of clinical oncology has shifted dramatically over the past two decades, with the rapid expansion of oral anticancer medications (OAMs) transferring the burden of treatment administration and toxicity monitoring from specialized infusion clinics to patients at home [1,2]. Historically, cytotoxic systemic therapy was predominantly delivered via intravenous (IV) infusions within closely monitored acute care or ambulatory hospital suites. Under that traditional framework, multidisciplinary oversight, acute toxicity monitoring, and administration safety were naturally centralized around healthcare professionals at the point of care [3].

However, the emergence of targeted oral oncolytics—including cyclin-dependent kinase 4/6 (CDK4/6) inhibitors, tyrosine kinase inhibitors (TKIs), and novel hormonal agents—has fundamentally altered this operational model by shifting the locus of care directly into patients’ homes [1,3]. While OAMs afford patients greater convenience and independence, they transfer the complex daily burden of treatment adherence, strict administration timing, food–drug interaction compliance, and early side-effect recognition entirely onto patients and their informal caregivers [4,5]. Consequently, outpatient non-adherence rates for OAMs can approach 50% in real-world populations, leading to therapeutic failure, premature treatment discontinuation, accelerated disease progression, and avoidable emergency department visits [6,7,8].

To bridge this outpatient care gap and mitigate medication-related risks, specialized ambulatory oncology clinical pharmacy services (CPSs) and protocol-driven toxicity management algorithms have become critical [9,10]. Dedicated clinical pharmacists perform vital, high-touch interventions, including Best Possible Medication History (BPMH) collection, comprehensive drug–drug interaction (DDI) evaluations, proactive toxicity triage, dose-adjustment management, and routine adherence support [4,11]. Evidence from landmark randomized controlled trials, such as the AMBORA study, has demonstrated that structured, clinical pharmacist-led care for patients on oral oncolytics yields statistically significant reductions in severe (CTCAE Grade ≥ 3) adverse drug events, decreases emergency hospitalizations, and improves treatment persistence [12].

However, an environmental scan of the Canadian practice landscape reveals significant systemic gaps and regional variability in the availability of ambulatory oncology clinical pharmacy services, with many centers facing severe human resource limitations [9,11]. As the volume and complexity of OAM regimens continue to surge, expanding specialized clinical pharmacy human resources through traditional full-time equivalent (FTE) clinical pharmacist hiring models remains difficult to execute sustainably within modern healthcare budget constraints [9,11].

**Figure 1:**
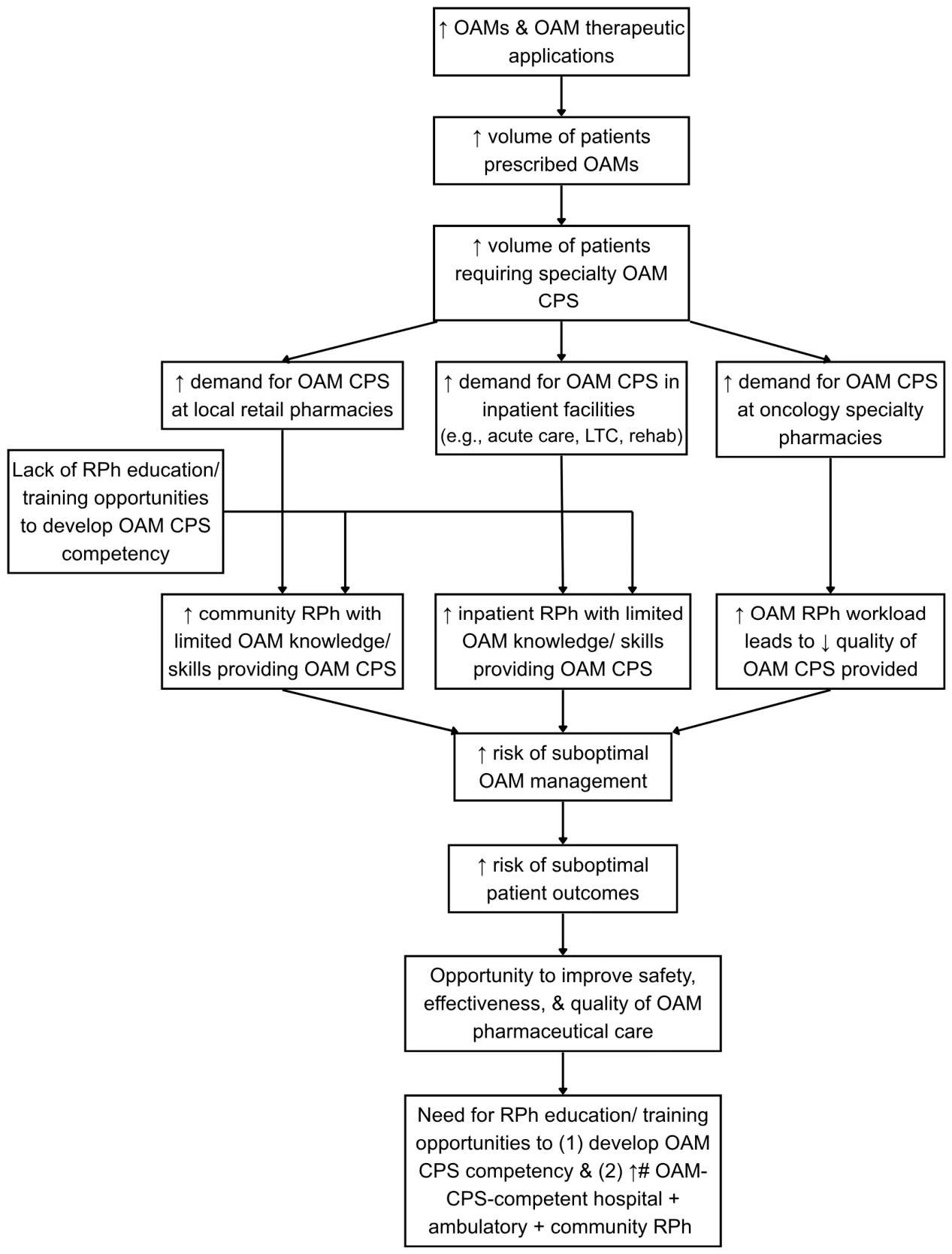
Flow chart showing relationship between rises in OAM use, increases in OAM CPS demand, & lack of OAM pharmacy education, which compromises the quality of OAM management and resulting treatment outcomes (OAM, oral anticancer medication; CPS, clinical pharmacy service; LTC, long term care; RPh, registered pharmacist; ↑, increased; ↓, decreased; ↑#, increased number).

Simultaneously, a persistent systemic educational gap exists across the pharmacy training landscape, compounding the pressure on oncology specialty services (Figure 1). A national survey of Canadian community pharmacists by Abbott et al. revealed that only 13.6% reported receiving adequate oncology education through their undergraduate training, continuing education, or community practice, and fewer than 10% felt comfortable educating patients on oral anticancer therapy [13]. These deficits persist in more recent data: a survey of Ontario community pharmacists demonstrated ongoing gaps in oncology-specific training, supporting the need for structured educational interventions [14]. Furthermore, oncology has been recognized as systematically under-represented in Canadian undergraduate and postgraduate training programs across pharmacy, medicine, and nursing [15].

As these training deficits intersect with surging OAM patient volumes, community and hospital pharmacists are increasingly forced to manage complex regimens with limited specialized knowledge, while specialty oncology pharmacists face unsustainable workload demands—a cascade that ultimately heightens the risk of suboptimal patient outcomes (Figure 1). While advanced experiential pharmacy trainees—including Doctor of Pharmacy co-op students and APPE interns—have been shown to successfully expand patient capacity, handle high-volume clinical task bundles, and generate documented cost savings across general ambulatory care clinics, outpatient settings, and Federally Qualified Health Centers [16,17,18,19], the formal integration of pharmacy trainees within specialized, high-acuity oral oncology ambulatory care programs remains under-reported in the published literature.

The deployment of pharmacy trainees to expand clinical capacity is not without operational debate. A recognized tension exists within health system management regarding the balance between operational task delegation and the supervisory time investment required of senior clinical staff [20]. Preceptor time costs are real and quantifiable, and, if these supervisory requirements exceed the clinical output generated by the trainee, the net departmental capacity gain may be limited [19,20].

Conversely, a systematic review of 29 published studies confirmed that pharmacy students in supervised experiential roles generally confer economic and clinical benefits exceeding the costs associated with their supervision and training [21]. When trainee activities are structurally mapped to established national benchmarking standards—such as Ambulatory Oncology Clinical Pharmacy Key Performance Indicators (AOcpKPIs) [11] or national inpatient cpKPI frameworks [22]—and governed by strict protocol-driven supervision, trainees can meaningfully contribute to core clinical workflows, thereby preserving senior pharmacist capacity for high-complexity clinical decision-making [19,21].

To address this dual operational capacity bottleneck and educational deficit, the Sunnybrook Odette Cancer Centre Pharmacy (SOCCP) designed and implemented the Oral Anticancer Medication Clinical Co-op Program (OAMCCP). This initiative built directly upon SOCCP’s established OAM clinical framework, which was recognized as an Accreditation Canada Leading Practice in 2017 [23] and awarded the 2023 Canadian Society of Hospital Pharmacists (CSHP) Excellence in Pharmacy Practice–Patient Care Award [24]. The primary objective of this pharmacist-led framework was to establish a structured, high-value practice learning pathway for PharmD co-op students while strategically adding net operational capacity to an already accredited OAM specialty workflow at a fraction of standard clinical pharmacist payroll costs, without compromising clinical quality or patient safety.

Accordingly, this study evaluated the multi-dimensional impact of the OAMCCP model over a multi-year period. Specifically, we quantified:

1. The clinical workload and service capacity generated by embedded co-op students;
2. The economic utility and cost efficiency ratio of student support relative to clinical pharmacist FTE compensation;
3. Patient-reported satisfaction and non-escalation rates of student-led encounters;
4. Qualitative educational value and clinical skill acquisition reported by participating trainees.

## 2. Materials and Methods

The institutional OAMCCP was formally launched in the fall semester of 2022. The operational design relied on onboarding one PharmD co-op student from the University of Waterloo School of Pharmacy per sequential 16-week academic term to work full-time alongside the SOCCP OAM team. A total of three students complete the rotation annually.

Each trainee received comprehensive standardized training and formative assessments to monitor ongoing progress, enabling real-time coaching to remediate skill gaps and ensure all learners met core competencies. The OAM team’s in-house assessment and management algorithms provided students with a consistent approach and structured framework to guide the timing of proactive follow-up encounters, adherence and toxicity parameters addressed at each touchpoint, suggested clinical assessment questions, toxicity grading scales, and evidence-based toxicity management strategies (e.g., non-pharmacological measures, pharmacological interventions, or physician escalation). The algorithms are specific to drug and disease site; for instance, the algorithm for afatinib in non-small-cell lung cancer (NSCLC) has been previously published in *Supportive Care in Cancer* [10].

During onboarding, students were trained to execute an array of clinical pharmacist duties. Initially, all patient-facing activities were directly observed by a supervising pharmacist. Once a student demonstrated clinical competence, they performed activities independently while escalating identified drug therapy problems (DTPs) or complex clinical issues to the supervising pharmacist. The operational and educational impact of the SOCCP OAMCCP was captured prospectively using a mixed-methods design across four key domains.

### 2.1. Workload and Service Capacity Analysis

Workload data were prospectively logged by two co-op students over a combined total of 15 weeks (6 weeks in fall 2022 and 9 weeks in summer 2023). Tracked clinical and administrative activities included: Best Possible Medication History (BPMH) completion, drug–drug interaction (DDI) screening, baseline counselling for OAMs and supportive care prescriptions, pre-encounter clinical chart reviews (e.g., evaluating laboratory parameters and progress notes prior to telehealth visits), proactive patient follow-up phone calls to assess adherence and manage toxicity-related DTPs, interprofessional communication of DTPs to prescribers, maintenance of the AOM team’s virtual care roster (auditing dispensary logs weekly to identify OAM initiations, confirm baseline services are complete, confirm addition to proactive call-back roster as per appropriate OAM algorithm), and maintenance of the team’s OAM reimbursement tracking database (receiving prior authorization response letters, updating the database and patient file accordingly).

The average number of tasks completed per week was calculated by dividing the total number of tasks logged by the total number of weeks in the evaluation period (15 weeks). The average weekly time investment was calculated by multiplying the average number of tasks completed per week by the approximate time investment required for each task in hours. Full-time equivalents (FTEs) were calculated by dividing the average weekly time investment by total hours per standard work week (37.5 h/week).

### 2.2. Economic Utility Analysis

Annual student payroll expenditure (44,700 CAD) was determined by summing costs across three academic terms (14,900 CAD/term). Full-time clinical pharmacist salary was benchmarked at CAD 161,200 inclusive of benefits. To determine the cost efficiency ratio of utilizing student support, the payroll cost for 1.0 PharmD student FTE was divided by the payroll cost for the projected pharmacist FTE equivalence.

### 2.3. Patient Satisfaction Evaluation

Patient satisfaction was evaluated by one co-op student during the summer 2023 term using a prospective convenience sample of 30 consecutive patients completing proactive telehealth follow-up calls. At the conclusion of the clinical encounter, consenting patients completed a brief feedback survey assessing: (1) overall satisfaction with student-provided care on a 10-point Likert scale (1 = very unsatisfied, 10 = very satisfied), and (2) if the student had successfully addressed all concerns by the close of the call.

### 2.4. Educational Value and Trainee Feedback

The first five PharmD co-op students completing the rotation (fall 2022–winter 2024) submitted qualitative reflections at the conclusion of their term. The OAMCCP lead reviewed these submissions to evaluate baseline expectations, clinical competency development, overall satisfaction, and perceived educational value of the experiential placement.

Generative artificial intelligence (Gemini 1.5 Pro, Google) was used to refine the language, improve readability, and assist with formatting this manuscript. The final text was fully reviewed, revised, and validated by the authors, who maintain complete accountability for this manuscript’s intellectual content.

## 3. Results

### 3.1. Workload and Service Capacity Analysis

OAMCCP trainees logged a cumulative total of 673 specialized tasks, averaging just under 45 individual interventions per week. A breakdown of specific tasks, hours invested, and estimated FTE equivalence is provided in Table 1. Proactive telephone follow-ups (214 completed interventions) and clinical chart reviews (279 completed interventions) consumed the largest portion of student time, accounting for an average of 5.71 h and 3.72 h per week respectively. Students contributed an average of 16.2 h of direct clinical support weekly, establishing a +0.43 FTE increase in real-time pharmacy department service capacity.

### 3.2. Economic Utility Analysis

Financial comparison highlighted a favorable health system economic outlook. Maintaining a full-time (1.0 FTE) clinical co-op student required a fixed operational payroll cost of approximately CAD 44,700 per annum. Given that the student absorbed workload equivalent to 0.43 FTE of a fully licensed ambulatory clinical oncology pharmacist—valued at an annual institutional baseline salary of CAD 69,300 for a 0.43 FTE portion—the model allowed the specialty care center to capture essential clinical hours for a fraction of traditional expenditures (CAD 44,700 for PharmD student 1.0 FTE/CAD 69,300 for Pharmacist 0.43 FTE = 65%).

### 3.3. Patient Satisfaction Evaluation

A cross-sectional cohort of 29 outpatients undergoing clinical management for multiple oncological agents (including lenvatinib, darolutamide, olaparib, and lenalidomide) completed satisfaction evaluations (Table 2). Since a total of 30 patients were approached to participate, this corresponds to an impressive 97% response rate. The mean clinical pharmacy service satisfaction score across the cohort was 9.5 out of 10. A total of 28 of 29 (96%) of patients surveyed confirmed that the embedded co-op student addressed all treatment-related questions and denied that further escalation to supervising pharmacist was required.

### 3.4. Educational Value and Trainee Feedback

**Figure 2:**
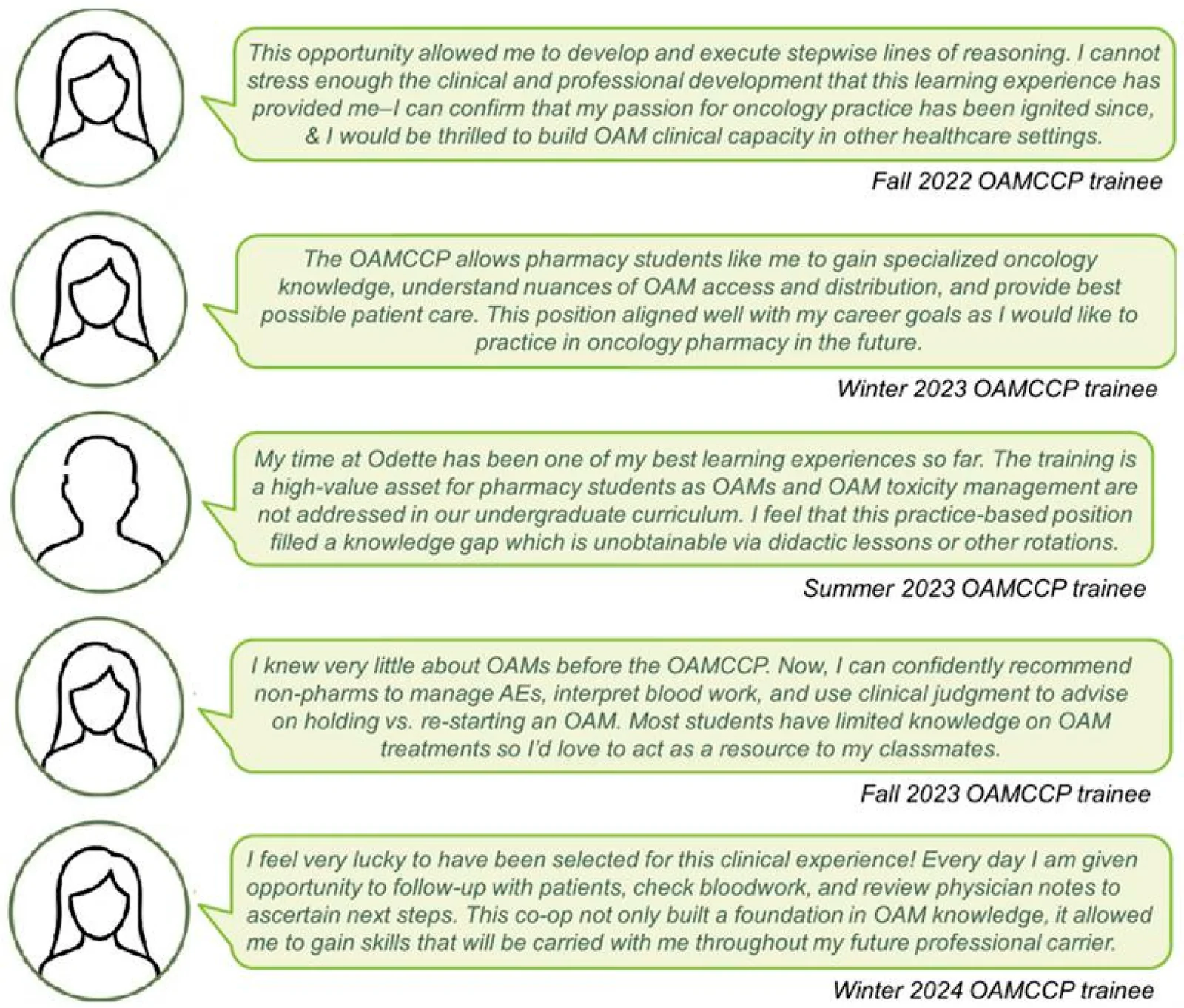
Select quotes from Oral Anticancer Medication Clinical Co-op Program (OAMCCP) students’ endorsement letters demonstrating the value and impact of the innovative learning opportunity.

Qualitative assessment of post-rotation portfolios suggested accelerated acquisition of complex professional knowledge. Select quotes from SOCCP OAMCCP trainee testimonials are provided in Figure 2. Trainees emphasized that the immersive framework successfully bridged gaps in didactic pharmacy education. Students reported acting as a clinical resource to peers, recognized the potential for OAM knowledge translation to the broader community, and felt that the transferrable knowledge/skills obtained during the co-op will help them provide high-quality pharmaceutical care in future practice.

## 4. Discussion

This prospective evaluation demonstrates that embedding a structured, specialized clinical co-op student model into an ambulatory oncology specialty pharmacy effectively expands service delivery capacity at a fraction of the cost of equivalent licensed pharmacist staffing. Over a 15-week observational window across two academic terms, PharmD co-op students completed 673 clinical pharmacist tasks (44.9 tasks/week), contributing 16.2 h per week of front line service. This equates to an estimated 0.43 FTE of added clinical capacity. Trainees operated within the SOCCP’s previously established, externally recognized OAM clinical framework [23,24], absorbing foundational processes—such as Best Possible Medication History (BPMH) collection and drug–drug interaction (DDI) screening, pre-encounter chart reviews, and adherence and toxicity outreach—which in turn enabled senior pharmacy staff to redirect time toward high-acuity drug therapy problems (DTPs) and complex management.

To ensure student activities reflected meaningful clinical care, the majority of tracked student duties were mappable to national consensus-derived Ambulatory Oncology Clinical Pharmacy Key Performance Indicators (AO-cpKPIs) [11]. BPMH collection, DDI screening, baseline OAM counseling, and DTP communication to the care team are mappable to AO-cpKPI 11, AOcpKPI 5, AOcpKPI 2, and AOcpKPI 12, respectively. As each follow-up encounter encompasses adherence assessment, toxicity assessment, and initiation of a pharmaceutical care plan, the tracked telehealth calls are mappable to AOcpKPI 13, AOcpKPI 14, AOcpKPI 8, and AOcpKPI 3 [11]. Aligning student activities with these standardized metrics demonstrates that learners actively contribute to core quality indicators linked to improved clinical outcomes. As emphasized by Lo et al. and Magedanz et al., institutional benchmarking must move beyond raw activity counts to capture discrete, quality-aligned process measures [25] and downstream clinical outcomes [26].

The clinical and economic impact of trainee-generated interventions observed is consistent with the broader ambulatory experiential literature [16,17,18,19,27]. Structured APPE programs in ambulatory and outpatient settings have consistently demonstrated that pharmacy trainees generate meaningful clinical outputs with high prescriber acceptance rates and documented economic value exceeding the costs associated with their supervision and training [21] [Mersfelder 2012].

In a study of cost savings from APPE interventions, Shepler [16] demonstrated that advanced pharmacy trainees generate substantial interventions correlating to quantified cost avoidance, averaging USD 148 per documented intervention. Similarly, Tualla et al. [18] demonstrated that embedding trainees within Federally Qualified Health Centers yields measurable clinical and economic value. At the program level, Woolley et al. documented USD 908,800 in cost avoidance across 87 students in a single academic year, with 44% of all interventions occurring in ambulatory care settings [17], and Stevenson et al. reported over 187,000 interventions and USD 6.2 million in cost avoidance across a 44-month school-wide APPE program, with nearly one-third of activity in outpatient and primary care environments [27]. Moreover, structured near-peer and layered-learning APPE models have expanded institutional rotation capacity up to 3.5-fold, with documented evidence from inpatient oncology units [19,28]. Our model corroborates these findings within a specialized ambulatory oncology setting, demonstrating that a 1.0 FTE student captures clinical hours valued at 69,300 CAD/year against a baseline payroll cost of 44,700 CAD/year—a cost-efficiency ratio of approximately 65%, representing approximately 35% gross payroll savings relative to equivalent licensed pharmacist staffing.

High patient satisfaction ratings (mean score 9.5 out of 10) suggest that integrating a learner maintained care delivery standards. Patients received timely telephone outreach that an otherwise resource-constrained staff might struggle to deliver consistently. Maintaining patient safety was central to this model. Supervision followed a tiered structure: every patient encounter was preceded by a pre-call briefing with the supervising pharmacist, and 100% of resulting documentation was reviewed and co-signed after the call. A more intensive post-call verbal debrief, along with real-time pharmacist input, was reserved for encounters in which a DTP was identified. This graduated approach ensures patient care standards are preserved across all encounters while concentrating the most intensive supervisory attention on the subset of cases carrying the greatest clinical risk.

Consistent with this framework, patients themselves reported needing escalation beyond the trainee’s own resolution in only 1 of 29 (3.4%) telehealth encounters, suggesting a high degree of patient-perceived issue resolution at the point of care. This is further corroborated by unsolicited, spontaneous positive feedback: over the program’s operational history—which will enter its 13th term in fall 2026—numerous patients have independently contacted the OAM team specifically to commend the quality of care provided by co-op trainees. The authors are also not aware of any reported adverse event, formal complaint, or identified clinical error attributable to trainee-delivered care. While these observations were not systematically tracked and are not a substitute for structured, quantified correction/escalation rate surveillance, the pattern of proactive, unprompted patient endorsement across multiple cohorts provides a real-world corroborating signal of safety and care quality alongside the formal survey results.

Furthermore, the OAMCCP also addresses a recognized oncology education gap across Canadian pharmacy programs [13]. Van der Linden and Van Aelst [29] emphasize that while the value of oncology pharmacists is clear, formal education in oncology pharmacotherapy remains a critical prerequisite for practice, describing it as a “conditio sine qua non for effective practice.” Dedicated oncology electives, such as the NCODA Oncology Basics Course described by Barash et al. [30], are increasingly explored to bridge entry-to-practice knowledge gaps, with demonstrated improvements in student pharmacist confidence and perceived competency in oncology topics. Our clinical co-op model provides immersive, experiential learning that equips trainees with specialized OAM competencies prior to graduation.

The rationale for prioritizing OAM clinical service expansion specifically stems from the unique vulnerabilities of self-administered oral cancer treatment. Suboptimal exposure driven by improper administration or non-adherence remains a major barrier to OAM efficacy [7,8]. Unlike intravenous regimens or monoclonal antibodies—which benefit from direct nursing administration and closed-loop facility oversight—self-administered OAMs shift the burden of adherence and toxicity management onto the patient with at-home therapies [4,5]. Furthermore, OAMs carry high drug interaction potential, with approximately 70% metabolized via the CYP3A4 pathway [31]. Robust randomized trial evidence from the landmark AMBORA trial [12] confirms that structured, proactive pharmacist-led OAM monitoring significantly reduces DTPs, improves adherence, and prevents avoidable emergency department visits.

### Limitations and Future Directions

Several limitations must be acknowledged. First, our figures were derived from two co-op students evaluated across 15 observational weeks at a single tertiary cancer center (*N* = 2). Student variations in clinical knowledge and efficiency may affect output; thus, the +0.43 FTE figure represents an initial estimate requiring validation in larger cohorts. Second, our economic model reflects gross captured clinical value and does not net out the time cost of student onboarding or daily pharmacist oversight.

Quantifying this supervisory burden directly—including the universal pre-call briefing and co-signature components alongside the conditional, DTP-triggered debriefing and real-time escalation components—remains essential for future net-cost evaluations. Third, although patient-requested escalation rates, spontaneous unsolicited patient endorsements, and the absence of known adverse events across the program’s operational history are all reassuring, this evaluation has two related but distinct gaps in its safety data. The rate at which the pharmacist’s pre-call briefing, post-call documentation co-signature, or DTP-triggered debrief identified an error, omission, or required modification to the student’s assessment or plan was not formally logged. This correction rate remains the more direct and important safety metric for a trainee delegation model and should be captured prospectively in future evaluations.

Separately, the frequency with which students sought real-time pharmacist input during an in-progress patient encounter—as opposed to issues identified only through the pre-call briefing, post-call co-signature, or post-call debrief—was also not tracked. This distinction may be clinically meaningful, as a need for real-time, mid-call intervention could indicate a higher-acuity encounter than one fully anticipated at pre-call briefing.

While under-reporting pharmacist input time to train and provide student oversight overestimates impact on workload capacity expansion, there were other factors that potentially underestimated student impact. For example, the list of student-tracked-tasks used for this project was not exhaustive. Notable non-tracked tasks, including documentation of patient care encounters in the electronic chart and responding to reactive patient enquires (as opposed to contacting patients for proactive follow-up), have a quantifiable impact on workload and were not explicitly tracked by trainees. Moreover, unobserved self-reported workload logs (data collection technique used in this study) may underestimate actual output. The work-sampling literature suggests healthcare staff report significantly fewer discrete clinical and non-clinical tasks during unobserved self-reporting compared to structured observational tracking or time studies [32].

An additional limitation is the use of a small convenience sample for the patient satisfaction survey (*n* = 29), which in turn may be subject to selection or social desirability bias. As with any voluntary survey, non-responders may differ systematically from responders, and the resulting sample may over-represent highly satisfied patients. Finally, our study did not explore delegating administrative or lower-acuity tasks to pharmacy technicians as an alternative strategy for expanding pharmacist capacity, which is another potential limitation. However, leveraging technician support was not feasible at our institution, where persistent technician retention challenges have precluded maintaining the stable technician workforce that such task delegation would require.

Future studies should evaluate applying structured quality improvement methodologies, including Lean Six Sigma approaches, to further enhance workflow standardization, operational efficiency, resource utilization, and the long-term sustainability of pharmacist-led OAM clinical workflows [33].

## 5. Conclusions

This single-site pilot demonstrates the feasibility of embedding a supervised PharmD co-op student into a specialized ambulatory OAM pharmacy program, providing an initial capacity expansion estimate of approximately 0.43 FTE (16.2 h/week). This capacity gain was achieved with no known safety compromises and, notably, with spontaneous, unsolicited positive patient feedback specifically praising trainee-delivered care across the program’s ongoing operational history. Aligned with national quality metrics (AO-cpKPIs) [11] and supported by standardized clinical algorithms [10,24], this operational model offers a structured framework for workforce integration. Whether these capacity expansion estimates and financial benefits generalize across diverse practice settings—and whether net economic benefits hold once supervising pharmacist time investments are fully quantified—requires confirmation in larger, multi-center evaluations using structured quality improvement methodology.

## Figures and Tables

**Table 1:** Co-op student workload performance over 15 weeks and projected capacity expansion in FTEs.

Clinical Service Metric	Total Tasks (Count)	Average Tasks per Week (Count)	Time per Task (Hours)	Time per Week (Hours)	Estimated FTE Equivalent
Best Possible Medication History	26.0	1.73	0.3	0.52	0.01
OAM & supportive care counseling	15.0	1.00	0.3	0.30	0.01
Drug-drug interaction screening	5.0	0.33	0.2	0.07	0.00
Clinical chart review	279.0	18.60	0.2	3.72	0.10
Proactive telehealth follow-up calls	214.0	14.27	0.4	5.71	0.15
Email communication to the care team	64.0	4.27	0.6	2.56	0.07
Management of clinical patient roster	31.0	2.07	1	2.07	0.06
Management of reimbursement tracking database	39.0	2.60	0.5	1.30	0.03
Totals	673.0	44.87	--	16.2	0.43

**Table 2:** Likert-scale responses to patient satisfaction survey. Patients rated their level of satisfaction with telehealth services on scale of 1–10.

Patient	Medication	Counseling Satisfaction
1	Lenvatinib	10
2	Darolutamide	8
3	Olaparib	10
4	Lenalidomide	10
5	Dabrafenib + Trametinib	10
6	Trametinib	4.5
7	Abiraterone	9
8	Osimertinib	10
9	Olaparib	9
10	Osimertinib	10
11	Capecitabine	10
12	Niraparib	10
13	Niraparib	10
14	Niraparib	10
15	Osimertinib	10
16	Olaparib	10
17	Niraparib	10
18	Niraparib	10
19	Niraparib	9
20	Lenalidomide	10
21	Regorafenib	10
22	Olaparib	8
23	Lenalidomide	10
24	Enzalutamide	10
25	Abiraterone	10
26	Niraparib	10
27	Enzalutamide	9
28	Alectinib	10
29	Enzalutamide	10

## Data Availability

The datasets generated and analyzed during the current evaluation are available from the corresponding author upon reasonable request, subject to institutional patient privacy restrictions.

## Abbreviations

The following abbreviations are used in this manuscript:

AOcpKPIs	Ambulatory oncology clinical pharmacy key performance indicators
APPE	Advanced pharmacy practice experience
BPMH	Best Possible Medication History
CPS	Clinical pharmacy services
DDI	Drug–drug interaction
FTE	Full time equivalent
OAM	Oral anticancer medication
OAMCCP	Oral Anticancer Medication Clinical Co-op Program
PharmD	Doctor of Pharmacy
SOCCP	Sunnybrook Odette Cancer Centre Pharmacy
